# A Dual Role of Complement Activation in the Development of Fulminant Hepatic Failure Induced by Murine-Beta-Coronavirus Infection

**DOI:** 10.3389/fcimb.2022.880915

**Published:** 2022-04-29

**Authors:** Yingying Fang, Yan Guo, Tongtong Gao, Xuelian Han, Yuting Jiang, Min Li, Wei Xue, Binhui Yang, Yujun Cui, Shihui Sun, Guangyu Zhao

**Affiliations:** ^1^ State Key Laboratory of Pathogen and Biosecurity, Institute of Microbiology and Epidemiology, Academy of Military Medical Sciences, Beijing, China; ^2^ School of Basic Medical Sciences, Anhui Medical University, Hefei, China

**Keywords:** betacoronavirus, MHV-3, complement, FHF, SARS-CoV-2, animal model

## Abstract

With the epidemic of betacoronavirus increasing frequently, it poses a great threat to human public health. Therefore, the research on the pathogenic mechanism of betacoronavirus is becoming greatly important. Murine hepatitis virus strain-3 (MHV-3) is a strain of betacoronavirus which cause tissue damage especially fulminant hepatic failure (FHF) in mice, and is commonly used to establish models of acute liver injury. Recently, MHV-3-infected mice have also been introduced to a mouse model of COVID-19 that does not require a Biosafety Level 3 (BSL-3) facility. FHF induced by MHV-3 is a type of severe liver damage imbalanced by regenerative hepatocellular activity, which is related to numerous factors. The complement system plays an important role in host defense and inflammation and is involved in first-line immunity and/or pathogenesis of severe organ disorders. In this study, we investigated the role of aberrant complement activation in MHV-3 infection-induced FHF by strategies that use C3-deficient mice and intervene in the complement system. Our results showed that mice deficient in C3 had more severe liver damage, a higher viral load in the liver and higher serum concentrations of inflammatory cytokines than wild-type controls. Treatment of C57BL/6 mice with C3aR antagonist or anti-C5aR antibody reduced liver damage, viral load, and serum IFN-γ concentration compared with the control group. These findings indicated that complement system acts as a double-edged sword during acute MHV-3 infection. However, its dysregulated activation leads to sustained inflammatory responses and induces extensive liver damage. Collectively, by investigating the role of complement activation in MHV-3 infection, we can further understand the pathogenic mechanism of betacoronavirus, and appropriate regulation of immune responses by fine-tuning complement activation may be an intervention for the treatment of diseases induced by betacoronavirus infection.

## Introduction

Betacoronavirus is an important group of human and animal pathogens, mainly causing respiratory and intestinal diseases (Nicklas et al., 2012). In recent years, the epidemic caused by betacoronavirus has killed tens of thousands of lives, especially the outbreak of COVID-19 in 2019 which has brought great disaster to people all over the world. And after the continuous emergence of SARS-CoV-2 mutants, it becomes more difficult to control the epidemic (Shi and Dong, 2021). Therefore, it is particularly important to have a deeper understanding of the pathogenic mechanism of betacoronavirus. However, for betacoronaviruses such as SARS-CoV-2, establishing pathological animal models without the need for biosafety level 3 (BSL-3) facilities remains a great challenge, which has largely hindered the understanding of the pathogenesis of SARS-CoV-2. The mouse hepatitis virus (MHV), which can work under Biosafety Level 2 (BSL-2), facilitates the study of the mechanism of immune damage caused by betacoronaviruses infection independent of BSL-3 conditions. MHV, a single-stranded RNA virus belonging to the genus betacoronavirus, is surrounded by protruding coronal spike proteins. MHV is antigenically related to a number of human types of coronavirus (Nicklas et al., 2012). The murine hepatitis virus strain-3(MHV-3) is the most virulent MHV and can cause fulminant hepatic failure (FHF) in susceptible mice (Belyavsky et al., 1998). MHV-3 infection in mice can reproduce the clinical symptoms of acute liver failure in FHF patients, thus, MHV-3-induced FHF can be used as a model to study the pathogenesis of viral hepatitis (Haring and Perlman, 2001). The latest study also shows that MHV-3 infection in mice can induce pathological manifestations similar to those of COVID-19 (Andrade et al., 2021). It can be seen that the study on the mouse model of MHV-3 infection contributes to a further understanding of the pathogenic mechanism of betacoronavirus, and meanwhile, it can also provide new ideas for the treatment of betacoronavirus infection.

FHF is a type of severe liver damage accompanied by multiorgan failure and an extremely high mortality rate. The causes of FHF vary and, in many patients, remain unclear (Bhaduri and Mieli-Vergani, 1996). Viral hepatitis is the primary etiopathogenic cause of FHF, accounting for more than 39% of patients with this disease (Liu et al., 2008). Mechanistically, local lesions can be triggered by various factors, including bacterial toxins, drugs, and free radicals (Kudo et al., 2009). However, the role of the complement system, which is closely associated with host immune responses, especially innate immune responses, in virus-induced FHF has not yet been clarified.

The complement system is a mediator between innate and adaptive immune responses and provides important first-line host defenses (Dunkelberger and Song, 2010). Complement could be activated *via* three conventional pathways: the classical, lectin, and alternative pathways. During the activation process, the C3 convertase cleaves C3 to C3a and C3b, and C3b is recruited to the C3 convertase to form the C5 convertase, which catalyzes cleavage of C5 to C5a and C5b to initiate the terminal complement pathway activation, resulting in the formation of the membrane attack complex (MAC), which plays an important role in the innate immunity. However, disturbances in the complement system are reported to contribute to the pathogenesis of various diseases (Hillebrandt et al., 2005; He et al., 2009; Roychowdhury et al., 2009; Ricklin et al., 2016). Depletion of complement from

mice reduced their morbidity and mortality rates despite increased viral replication and spread, suggesting that complement may play a pathologic role in virus-induced diseases (Hirsch et al., 1980). After treatment with C5a receptor antagonist (C5aRa), the difference in inflammatory response and liver injury between MHV-59A infected wild type mice and Msr1-/- mice decreased, indicating that C5a-induced pro-inflammatory response played a key role in the pathogenesis of Msr1-mediated liver injury (Tang et al., 2018). Administration of CD55, a regulator of the complement pathway, efficiently reduced inflammation, steatosis, and fibrosis in the livers of transgenic mice conditionally expressing HCV core protein (Chang et al., 2009). More recently, Liu j et al. (2015) demonstrated that TNF-α, C5aR and fibrinogen-like protein 2 (FGL2) contribute to coagulation in virus-induced fulminant hepatitis. Taken together, these findings indicated that aberrant complement activation plays a role in virus-induced liver injury.

To investigate systematically the role of complement in betacoronavirus infection, a mouse FHF model was established in this study by challenging mice with MHV-3. The effect of depletion of complement components on FHF caused by betacoronavirus infection was assessed.

## Materials and Methods

### Animals and Materials

Wild-type (wt) female C57BL/6, and C3-deficient (C3-/-; B6.129S4-C3tm1Crr/J) mice aged 8 weeks were obtained from the Jackson Laboratory. The C3aR antagonist SB 290157 was purchased from Calbiochem (San Diego, CA, USA). The monoclonal Ab (mAb) against mouse C5aR (HM1076, clone20/70) and the isotype Rat IgG2b (HI4041, clone RTK4530), were purchased from Hycult Biotech, The Netherlands.

### MHV-3

MHV-3 was plaque-purified and expanded in the 17Cl-1 cell line. After about 3-4 days, the virus-containing supernatants were collected and stored at -80°C until use. Each mouse was intraperitoneally injected with 100 PFU MHV-3 in a total volume of 200 µl.

### Animal Experiment

Five groups (with three to five animals in each group) of mice were infected with MHV-3 (Nicklas et al., 2012): wt C57BL/6 mice (Shi and Dong, 2021); C3-deficient mice (C3-/- group) (Belyavsky et al., 1998); wt C57BL/6 mice intravenously injected with 1 mg/kg C3aR antagonist 12 and 36 hrs after MHV-3 infection (C3aRa group); and (4 and 5) wt C57BL/6 mice intravenously injected with a monoclonal antibody (mAb) specific for C5aR (C5aR Ab group) or its isotype 12 hrs after MHV-3 infection.

To detect complement activation, wt mice were euthanized 0, 6, 12, 24, and 48 hrs after MHV-3 infection. Serum samples were collected and FUT-175 were added to serum samples at the time of collection to provide additional protection from complement ex vivo activation, and concentrations of C3a, C5a, liver enzymes, and proinflammatory cytokines were measured. Liver tissues were processed for the deposition of C3 and C5b-9 and the expression of C3aR and C5aR by immunohistochemistry, and for levels of C3aR mRNA and C5aR mRNA by RT-qPCR.

All other groups of animals were euthanized 24 and 48 hrs after MHV-3 infection. Liver enzymes and proinflammatory cytokines were measured at the indicated time. Liver tissues were processed for histopathological analysis and virus antigen expression detection. The viral titers in liver tissue samples were determined by the tissue culture infectious dose (TCID_50_) method, and was calculated by the Reed and Muench method and expressed as log_10_ 50% tissue culture infectious dose (TCID_50_)/g of liver tissues.

### Histological Analysis and Immunohistochemical Staining

Liver tissue samples were fixed in 10% formalin at room temperature, embedded in paraffin, and sliced into sections, which were stained with hematoxylin and eosin (H&E). The extent of liver damage in each sample was assessed by two independent observers blinded to the treatment groups.

Six micrometer sections of formalin-fixed, paraffin-embedded liver tissue specimens were deparaffinized, and antigen was retrieved with the microwave method. The sections were incubated overnight at 4°C with monoclonal antibodies specific for C3 (HM1045), C3aR (H-300), CD88 (H-100), and neutrophils (NIMP-R14) (Santa Cruz Biotechnology, Inc. California), anti-CD68 antibody (ab125212, Abcam Inc, MA, USA) and C5b-9 (Calbiochem, Germany), and with mouse anti-MHV polyclonal antibody (pAb; generated in mouse). The antibody isotypes were used as controls. The sections were washed and incubated with biotinylated secondary antibody, followed by an avidin-biotin-peroxidase conjugate (Beijing Zhongshan Biotechnology Co., Ltd. China). Immunoreactivity was detected using DAB and by counterstaining with hematoxylin.

### Measurement of Proinflammatory Cytokines in Serum

Serum concentrations of alanine aminotransferase (ALT) were measured using a Beckman CX5 Chemistry Analyzer, and serum concentrations of the proinflammatory cytokines interleukin (IL)-6, tumor necrosis factor (TNF)-α, and interferon (IFN)-γ were measured by ELISA kit (eBioscience, Vlenna, Austria) according to the manufacturer’s instructions.

### Relative Quantitative Real-Time PCR

Total RNA was isolated from liver tissue and RT-qPCR was performed using primers for C3aR, C5aR and GAPDH (Sun et al., 2013). The primers for C3aR were 5’-tctcactgaggcatctattcagtt-3’ (sense) and 5’-attgccgtgctacgttctg-3’ (antisense). The primers for C5aR were 5’-cgctcatcctgctcaacat-3’ (sense) and 5’-acggtcggcactaatggtag-3’ (antisense). The primer for GAPDH were 5’-CTCTGACTTCAACAGCGACAC-3’ (sense), 5’-CGTTGTCATACCAGGAAATGA-3’ (antisense). The relative levels of C3aR and C5aR mRNAs were determined using the 2^-ΔΔCT^ method (Livak and Schmittgen, 2001).

### Statistical Analysis

All statistical analyses were performed using the Graphpad Prism Program (version 7.00; GraphPad Software, Inc.). The concentrations of inflammatory cytokines and ALT at 24 and 48 hrs, viral titers, and neutrophil infiltration were compared using Student’s t test with Welch’s correction. Results at different time groups were analyzed by one-way ANOVA with Dunnett’s post test. Between-group differences in viral titers and ALT at the indicated times were analyzed by two-way ANOVA with Bonferroni post test.

## Results

### Complement Activation in Mice After MHV-3 Infection

**Figure 1:**
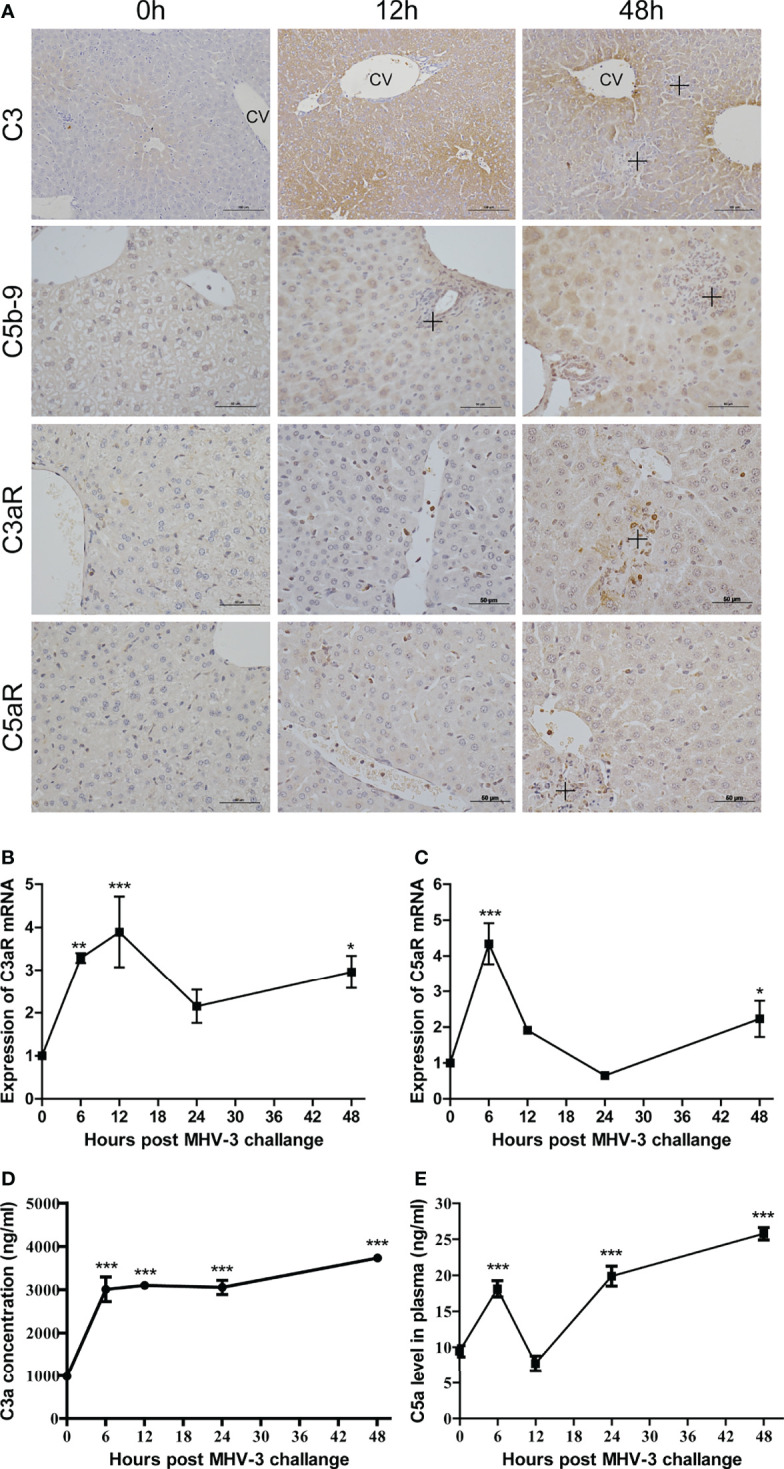
Involvement of complement after MHV-3 infection. WT mice were euthanized 0, 6, 12, 24, and 48 hrs after MHV-3 challenge. **(A)** Immunohistochemical staining for the deposition of C3, C5b-9, and for the expression of C3aR and C5aR in liver tissue samples (CV, central vein; cross, inflammatory infiltrates or necrotic areas). **(B, C)** Levels of C3aR mRNA and C5aR mRNA, as determined by relative quantitative real-time RT-qPCR in liver tissues at the indicated times. **(D, E)** Serum concentrations of C3a and C5a at the indicated times. These results are representative of three independent experiments with similar results (n = 4–5 per group). *p<0.05, **p<0.01, ***p<0.001 compared with 0 time. C3 original magnification, ×200; C5b-9, C3aR, and C5aR original magnification, ×400.

The activation of complement after MHV-3 infection was assessed by measuring the amounts of C3 and C5b-9 deposited in liver tissues. There is only slight C3 deposition around the central vein (CV) at 0 hr, but viral infection increased C3 deposition in the parenchyma, particularly around the CV and in necrotic areas, at 12–48 hrs (**Figure 1A**). Similarly, the deposition of C5b-9 was also increased after MHV-3 infection.

Expression of C3aR and C5aR in liver tissue was analyzed by relative quantitative real-time RT-qPCR and immunohistochemistry, respectively. At 0 hr, C3aR and C5aR proteins were expressed mainly on Kupffer cells, with their expression increasing over time; at 48 hrs, C3aR and C5aR proteins were also expressed on some hepatocytes and inflammatory cells, particularly in areas of necrosis (**Figure 1A** and **Supplementary Figure 3**). The levels of C3aR and C5aR mRNAs increased soon after infection and peaked at 6 and 12 hrs, respectively. Although their levels decreased at 24 hrs, a second phase of expression peaks at 48 hrs (**Figures 1B, C**). The serum concentrations of C3a and C5a increased until 48 hrs, although there was a reduction in C5a concentration 12 hrs after viral infection (**Figures 1D, E**). These findings showed that infection with MHV-3 resulted in complement activation systemically and in mouse livers.

### Severe Pathologic Damage and High Virus Replication and Inflammatory Responses in C3-Deficient Mice

**Figure 2:**
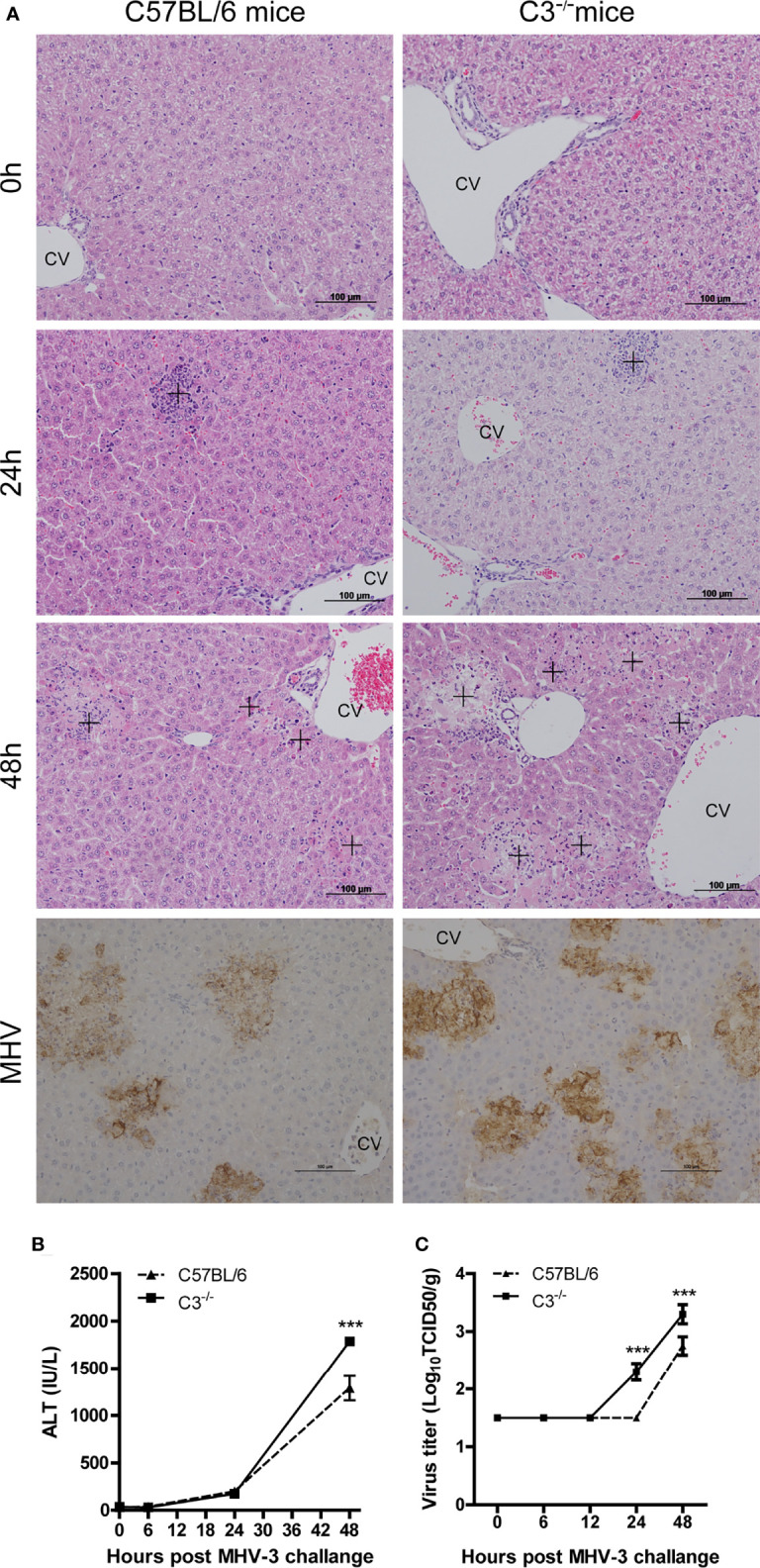
Severe liver damage in C3-deficient mice after MHV-3 infection. WT and C3-/- mice were euthanized 0, 6, 12, 24, and 48 hrs after MHV-3 challenge. **(A)** Histological examination of liver tissue of mice at the indicated times and immunohistochemical staining for viral antigen in liver samples 48 hrs after virus challenge (cross, inflammatory infiltrats area in 24hrs and necrotic area in 48hrs). **(B)** Serum ALT concentrations at the indicated times. **(C)** Virus titer in livers at the indicated times. These results are representative of three independent experiments with similar results (n = 4–5 per group). ***p<0.001 compared with WT control. Original magnification, ×200.

Increased complement activation is closely associated with the aggravation of diseases caused by SARS-CoV-2 infection (Holter et al., 2020), and we hypothesized that complement activation was associated with histopathological damage in MHV-3-challenged mice. To confirm our hypothesis, C3-/- and wt mice were challenged with MHV-3 and livers were collected at the indicated time points. There was no apparent difference in liver damage between C3-/- and wt mice at 12 and 24 hrs after MHV-3 infection. However, liver damage was more severe in C3-/- than in wt mice 48 hrs after MHV-3 infection (**Figure 2A** and **Supplementary Figure 4A**), a finding confirmed by the higher serum ALT concentration in C3-/- mice 48 hrs after MHV-3 challenge (**Figure 2B**). To further assess the relationship between liver damage and viral replication, we analyzed MHV-3 replication and viral antigen expression. The replication of virus was higher in C3-/- than that in wt mice, as detected by immunohistochemical staining and TCID50 (50% tissue culture infectious dose) analysis (**Figures 2A, C**).

**Figure 3:**
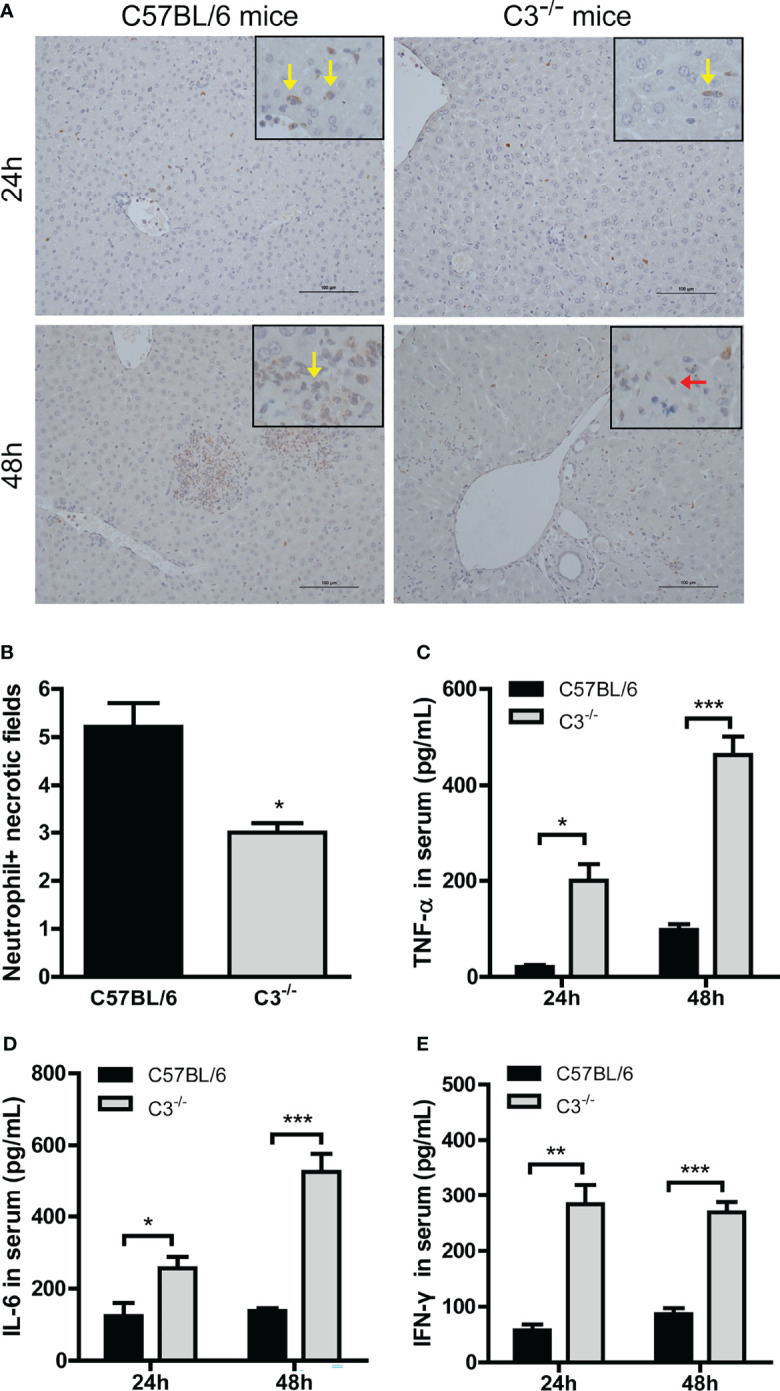
Higher inflammatory responses in C3-deficient mice after MHV-3 infection. Serum and liver samples were obtained from WT and C3-deficient mice euthanized 24 and 48 hrs after MHV-3 challenge. **(A)** Immunohistochemical staining for neutrophil infiltration in liver samples (yellow arrow, infiltrated neutrophil; red arrow, necrotic hepatocytes). **(B)** Semiquantitative assessment of neutrophil infiltration in livers 24 hrs after virus infection. **(C–E)** Serum concentrations of proinflammatory cytokines 24 and 48 hrs after MHV-3 challenge. These results are representative of three independent experiments with similar results (n = 4–5 per group). *p<0.05, **p<0.01, ***p<0.001 compared with WT control. Original magnification, × 200.

Neutrophil infiltration was lower in C3-/- than in wt mice at 24 and 48 hrs after MHV-3 infection (**Figures 3A, B**), indicating that the normal inflammatory response had a protective role against virus infection. Serum concentrations of TNF-α, IL-6, and IFN-γ were higher in C3-/- than in wt mice 24 and 48 hrs after infection (**Figures 3C** **–E**), indicating that mice genetically deficient in C3 may experience more severe liver damage due to the greater replication of viral load.

### C3aR Antagonist Alleviates Liver Damage in Mice Infected with MHV-3

**Figure 4:**
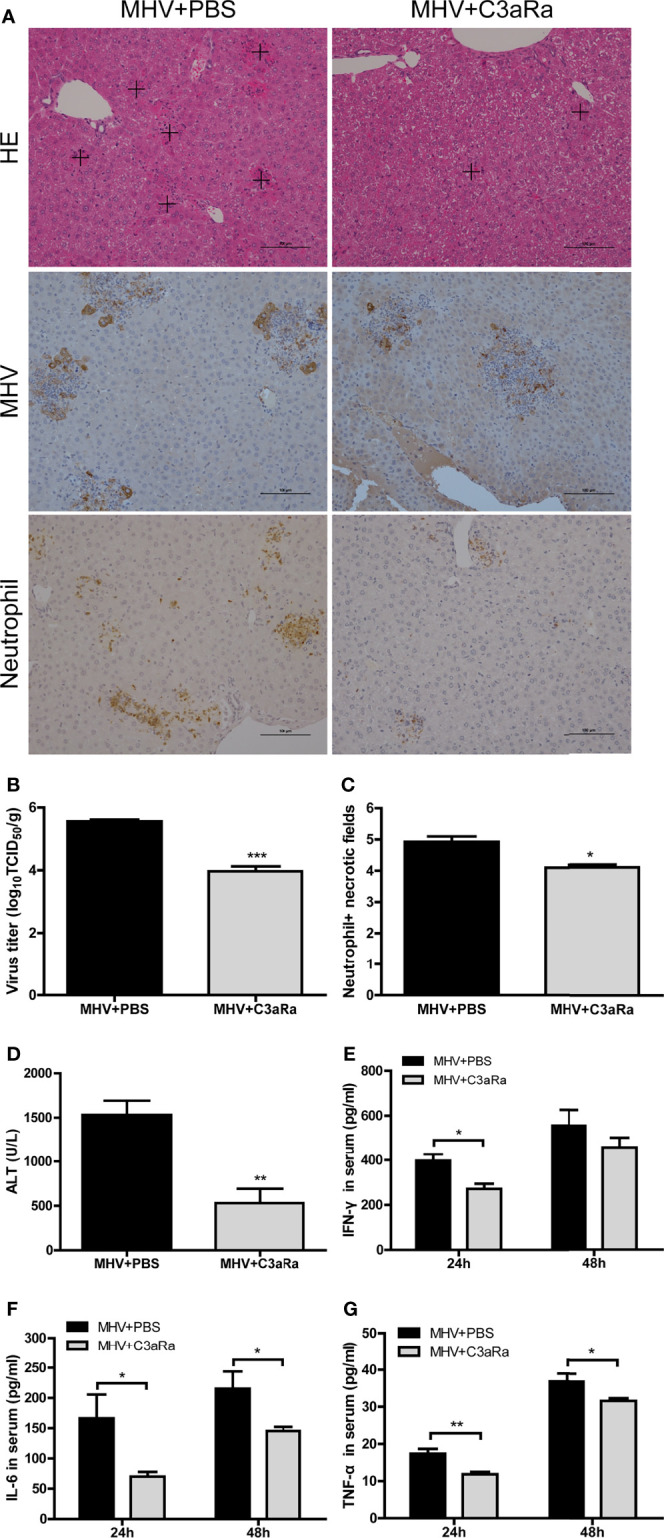
Attenuated liver damage in mice treated with C3aR antagonist after MHV-3 infection. Mice were administered C3aR antagonist 12 and 36 hrs after MHV-3 infection and euthanized, and liver tissue and serum samples were collected. **(A)** Histological examination of liver tissue, and immunohistochemical staining for viral antigen and neutrophil infiltration in mouse livers 48 hrs after virus infection (cross, multifocal damage area). **(B)** Virus titer in livers 48 hrs after virus infection. **(C)** Semiquantitative assessment of neutrophil infiltration in livers 48 hrs after virus infection. **(D)** Serum ALT concentrations 48 hrs after virus infection. **(E–G)** Serum concentrations of proinflammatory cytokines 24 and 48 hrs after MHV-3 infection. These results are representative of three independent experiments with similar results (n = 4–5 per group). *p<0.05, **p<0.01, ***p<0.001 compared with WT control. Original magnification, × 200.

The results observed in infected C3-/- confirmed that the complement system protects against liver injury after viral infection. However, complement activation products C3a and C5a are potent inflammatory mediators, which have close relations with host immune responses by binding to their receptors C3aR and C5aR (Klos et al., 2009). To assess the effect of blocking C3a and C3aR binding in liver damage after viral infection, we analyzed immune responses and virus replications in mice administered C3aR antagonist 12 and 36 hrs after MHV-3 infection. Liver damage was less severe in mice treated with C3aR antagonist than untreated wt mice, with reduced neutrophil and macrophage infiltration in the liver (**Figures 4A, C** and **Supplementary Figures 1, 4B**), lower serum ALT concentration (**Figure 4D**), lower expression of viral antigen (**Figure 4A**), lower viral titer in the liver (**Figure 4B**), and lower serum concentrations of inflammatory cytokines, especially TNF-α and IL-6 (**Figures 4E–G**), as assayed at 48 hrs after MHV-3 infection.

### Anti-C5aR Antibody Treatment Alleviates Liver Damage in Mice Infected with MHV-3

**Figure 5:**
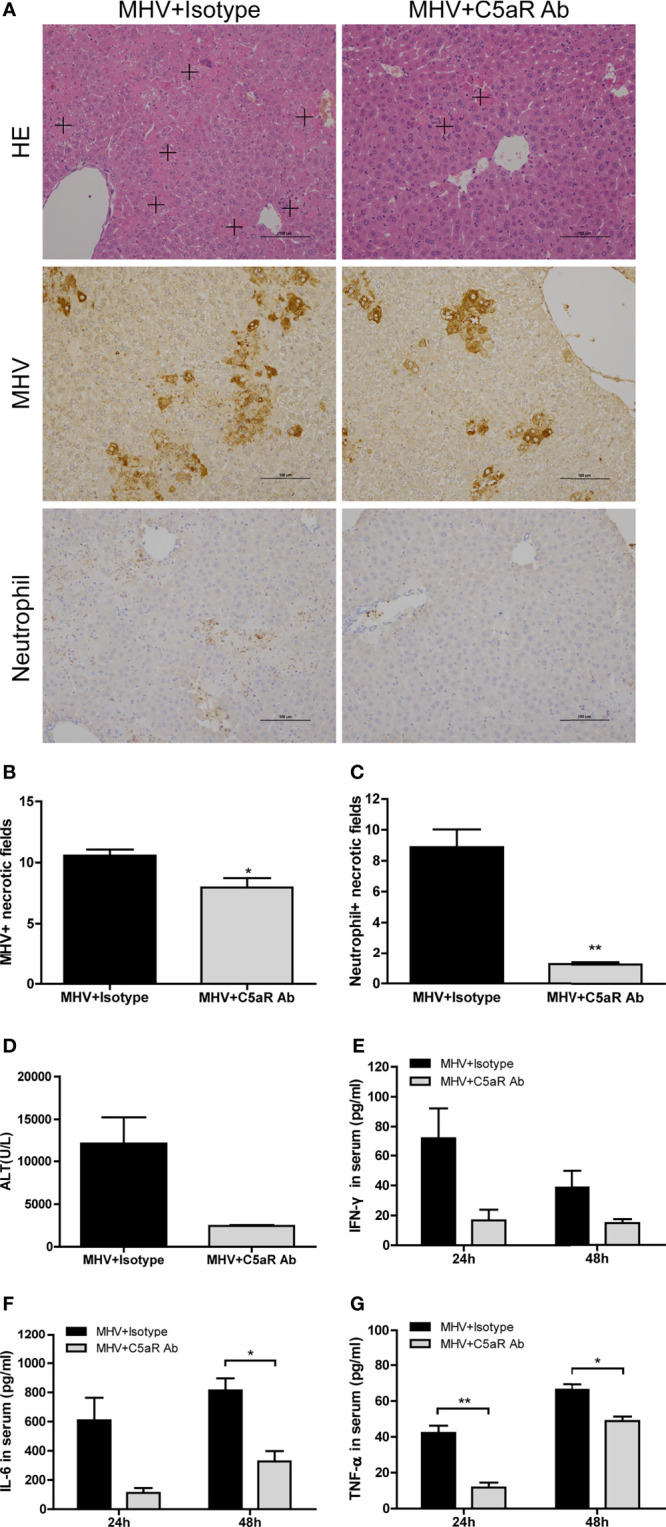
Attenuated liver damage in mice treated with anti-C5aR antibody after MHV-3 infection. Mice were administered anti-C5aR antibody or the isotype antibody 12 hrs after MHV-3 infection and euthanized, and liver tissue and serum samples were collected. **(A)** Histological examination of liver tissue, and immunohistochemical staining for viral antigen and neutrophil infiltration in mouse livers 48 h after virus infection (cross, multifocal damage area). **(B, C)** Semiquantitative assessment of viral antigen and neutrophil infiltration in livers 48 hrs after virus infection. **(D)** Serum ALT concentrations 48 hrs after virus infection. **(E–G)** Serum concentrations of proinflammatory cytokines 24 and 48 hrs after MHV-3 challenge. These results are representative of three independent experiments with similar results (n = 3-4 per group). *p<0.05, **p<0.01 compared with the isotype control. Original magnification, × 200.

**Figure 6:**
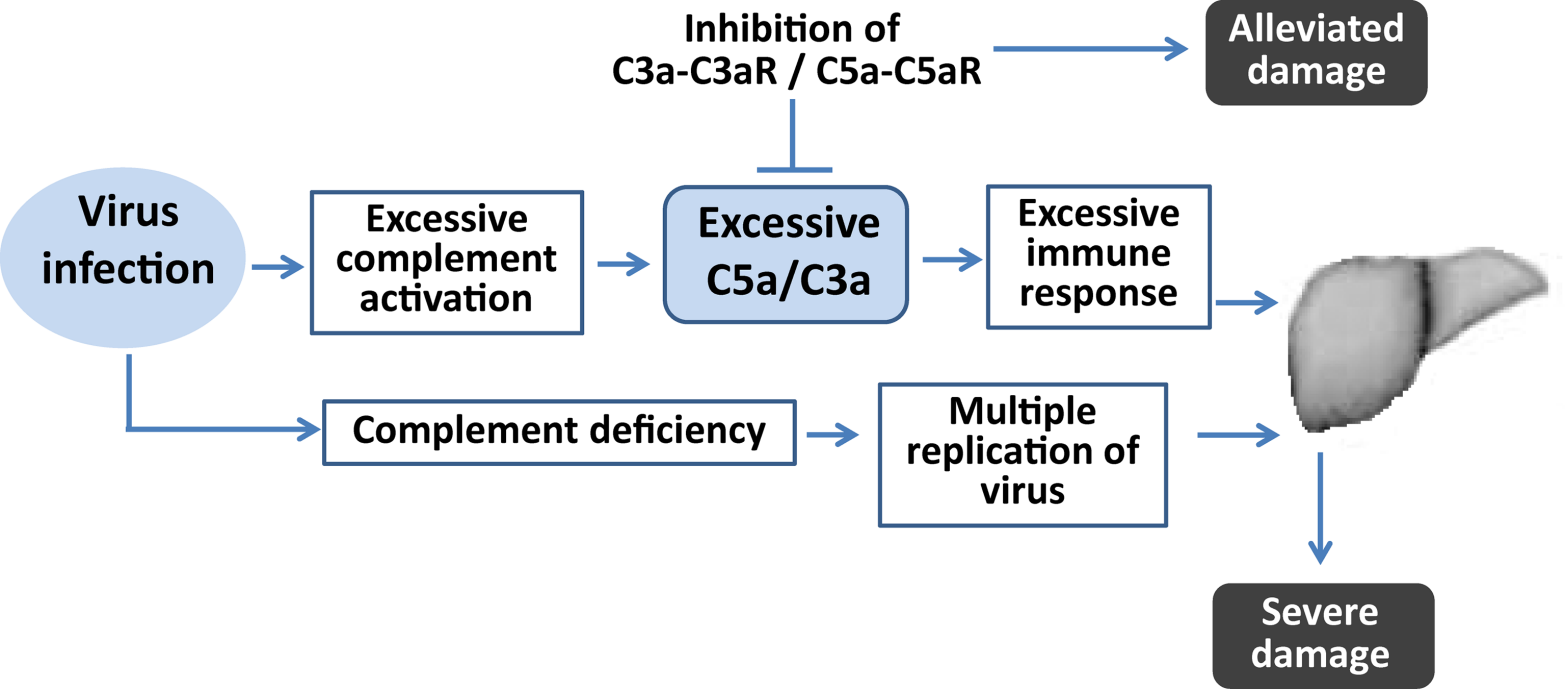
Diagram illustrating the role of complement in virus-induced fulminant hepatic failure. Complement deficiency contributes to more severe liver damage because of uncontrollable viral replication. Sustained complement activation after virus infection contributes to excessive inflammatory responses. Regulation of the complement by inhibition of its activation could alleviate liver damage by regulating the imbalanced inflammatory response.

To further assess the effect of blocking C5a and C5aR binding in liver injury after viral infection, we analyzed immune responses and virus replication in mice treated with anti-C5aR Ab after MHV-3 infection. Liver damage, viral antigen expression, and neutrophil and macrophage infiltration in the liver (**Figures 5A–C** and **Supplementary Figures 2, 4C**) were lower after anti-C5aR Ab treatment. In addition, mice treated with anti-C5aR Ab had lower serum concentrations of ALT (**Figure 5D**) and inflammatory cytokines, especially TNF-α and IL-6 (**Figures 5E–G**), 48 hrs after MHV-3 infection, consistent with our prior observations made by administrating C3aR antagonist. Dummy **Figure 6.**

## Discussion

This study showed that mice deficient in C3 experienced more severe liver damage and a higher viral load in the liver than wt mice. Moreover, regulation of complement systems during the course of infection by administration of C3aR antagonist or anti-C5aR Ab could limit virus proliferation and attenuate liver damage. These results indicate that complement activation plays an important role in defending against viral infection, whereas the anaphylatoxins C3a and C5a produced by complement activation after virus infection mediate harmful inflammation, leading to severe liver damage. Regulating inflammatory responses by targeting complement may therefore be an effective clinical intervention and adjunctive treatment in patients with betacoronavirus-induced diseases.

Zoonoses have been a major challenge to global public health, and they account for the majority of emerging and re-emerging infectious diseases (Dong and Soong, 2021). COVID-19 is a recent zoonotic disease that poses a great threat to human health and has become a global epidemic, and the research on betacoronaviruses including SARS-CoV-2 is becoming increasingly important. SARS-CoV, MERS-CoV and SARS-CoV-2 infections mainly cause damage to the respiratory system. However, during the follow-up of clinical patients, Cai et al. found that 76.3% of the patients had abnormal liver examination results during hospitalization, and 21.5% of the patients had liver injury (Cai et al., 2020). In the clinical analysis of SARS-CoV infection, it is also found that the liver damage induced by SARS seems to be caused by SARS virus directly (Yang et al., 2005). In our previous study, MERS-CoV infection in transgenic mice caused a large number of macrophage infiltration in liver tissue and hepatocyte necrosis (Yang et al., 2005; Zhao et al., 2015). It can be seen that liver injury is also a reason for the aggravation of the disease after coronavirus infection. Our results reveal that complement plays a double-edged role in liver injury caused by MHV-3 infection. On the one hand, the normal activation of complement will protect the body against virus, and on the other hand, abnormal activation of complement will lead to the aggravation of inflammation, resulting in acute liver injury.

MHV, like SARS-CoV-2, belongs to betacoronavirus genus. Because of its different organ tropism, it provides a great experimental platform for understanding the pathobiology of respiratory tract, liver and nervous system (Weiss and Leibowitz). MHV-A59 and MHV-1 can cause pulmonary infection and clinical manifestations of severe pneumonia in mice, which can be used as a potential model for the study of highly pathogenic betacoronavirus (De Albuquerque et al., 2006; Yang et al., 2014). Neurotropism of MHV is a potential model for studying COVID-19 neurological diseases (Chakravarty and Das Sarma, 2021). Recently, MHV-3-infected mice were introduced into COVID-19 mouse model, which has very similar clinical manifestations to COVID-19 patients (Andrade et al., 2021). MHV-3 is the most virulent of MHV and can cause liver damage in susceptible mice. It has been found that MHV-3 can induce prothrombinase reaction in mice through the induction of monocyte/macrophage procoagulant activity, which is the vital cause of FHF (Levy et al., 1981). Our study shows that the continuous activation of complement plays a crucial role in inflammatory reaction, which may also be a factor of liver injury caused by MHV-3 infection. This may also be a cause of the pathogenicity of human coronavirus.

The complement system plays an essential role in the innate immune responses *via* regulation of inflammatory responses and participates in the recognition and elimination of pathogens *via* various mechanisms (Moulton et al., 2008; O’Brien et al., 2011). In the present study, liver injury was found to be worse in C3-deficient mice, being accompanied with greater virus replication in the liver when compared with wild type mice. As C3 are central components in complement system, deficiency in C3 may result in a compromised immune system to the process of viral clearance thereby increasing viral titers. It is possible that enhanced cytopathic effects (CPE) due to increased viral loads in C3-deficient mice are the main mechanism for the liver injury. Intriguingly, blockade of downstream proinflammatory signaling pathway of complement activation during the viral infection in mice resulted in completely different outcomes. Accumulated studies indicated that despite the importance in recognizing and eliminating pathogens, the complement system is also involved in the deterioration of diseases caused by highly pathogenic betacoronavirus. For example, the complement components in SARS-CoV-2 patients are higher than those in mild patients (Holter et al., 2020). MERS-CoV infection-induced complement overactivation may lead to apoptosis and inflammatory responses in hDPP4 transgenic mouse model (Jiang et al., 2018). Our previous studies also demonstrated that excessive complement activation appears to exacerbate viral infection through the dysregulation of host immune response (Sun et al., 2013; Sun et al., 2015). In this model of FHF, activation of the complement system is sustained after viral infection, similar to findings in patients with FHF (Pham et al., 1995). Treatment of mice with C3aR antagonist or anti-C5aR Ab alleviated liver damage and dysregulated immune responses, which indicated that FHF observed in wt mice would partially be the result of overwhelming activation of complement. Therefore, complement activation might play a protective role during early stages of viral infection, but its sustained activation may mediate disordered inflammatory responses, and induce severe liver damage.

The liver is a lymphoid organ composed of hepatocytes, Kupffer cells, NK cells, and NKT cells, which may be activated by pathogen invasion and respond to inflammatory cytokines (Crispe, 2009). IFN-γ is an effector cytokine produced mainly by Th1 cells, CD8+ T cells, and NK cells, the expression of which may be modulated by complement activation (Yalcindag et al., 2006; Lalli et al., 2007; Baruah et al., 2009; Chauhan and Moore, 2011; Fusakio et al., 2011). Although the role of IFN-γ in FHF is still unclear, IFN-γ may negatively regulate IL-17-mediated immunopathology during hepatitis viral infection in a MHV-A59 virus-infected IFN-γ-deficient mouse model (Yang et al., 2011). Other studies, however, suggest that expression of IFN-γ is closely associated with liver injury such as fulminant hepatitis B, acute or chronic liver failure, and FHF-associated hepatocellular injury (Kimura et al., 1999; Zou et al., 2009; dos Santos et al., 2012). Similarly, in SARS-CoV-2 infection, TNF-α and IFN-γ can jointly induce cell death. Therefore, IFN-γ plays an important role in virus infection. The present study showed that IFN-γ concentrations decreased and liver injury was alleviated after treatment with C3aR antagonist or anti-C5aR Ab following viral infection. These findings indicated that complement-regulated IFN-γ secretion played a vital role in virus-induced FHF.

In conclusion, this study showed that the complement system is highly involved in FHF after MHV-3 infection. The dysregulated activation of complement system leads to sustained inflammatory responses and induces extensive liver damage. In addition, MHV is a good model for studying the pathogenesis of betacoronavirus immune response. Compared with SARS-CoV and SARS-CoV-2, MHV requires only BSL-2 but not BSL-3 containment. Although MHV may not be able to fully summarize the physiological characteristics of betacoronavirus, MHV can provide some insights into the viral biology of betacoronavirus, especially our research can provide some data for complement activation in betacoronavirus.

## Data Availability Statement

The original contributions presented in the study are included in the article/**Supplementary Material**. Further inquiries can be directed to the corresponding authors.

## Ethics Statement

All procedures involving animals were approved by the Institutional Animal Care and Use Committee (IACUC) of the Laboratory Animal Center, State Key Laboratory of Pathogen and Biosecurity, Beijing Institute of Microbiology and Epidemiology (Permit Number BIME 2017-15).

## Author Contributions

SS and GZ conceived and designed this study. YF and XH wrote the first draft of the article and finalized it. YG and TG performed the experiments, and XH and YJ completed analysis and interpretation the data. ML, WX, BY and YC make critical comments on the manuscript. YC, SS and GZ revised the article. All authors contributed to the article and approved the submitted version.

## Funding

This work was supported by grants from the Independent Fund of the State Key Laboratory of Pathogen and Biosecurity (SKLPBS2101), the National Natural Science Foundation of China (82102369), the National Key Plan for Scientific Research and development of China (2016YFD0500306), the National Project of Infectious Diseases (2017ZX10304402-003).

## Conflict of Interest

The authors declare that the research was conducted in the absence of any commercial or financial relationships that could be construed as a potential conflict of interest.

## Publisher’s Note

All claims expressed in this article are solely those of the authors and do not necessarily represent those of their affiliated organizations, or those of the publisher, the editors and the reviewers. Any product that may be evaluated in this article, or claim that may be made by its manufacturer, is not guaranteed or endorsed by the publisher.
