# Supplementary material for: A Dual Role of Complement Activation in the Development of Fulminant Hepatic Failure Induced by Murine-Beta-Coronavirus Infection
Source: Front Cell Infect Microbiol. 2022 Apr 29;12:880915. doi: 10.3389/fcimb.2022.880915 (PMC9099255; doi:10.3389/fcimb.2022.880915)
Supplement: Supplementary file 1 [file DataSheet_1.docx]

Supplementary Material

# Supplementary Methods

## Semiquantitative assessment of complement composition and liver injury

For semiquantitative assessment of the deposition of C3 and C5b-9. 10× objective fields of liver parenchyma in each liver section were examined by light microscopy for the deposition of C3, C5b-9. The scores for each animal were calculated according to the percentage of positive fields accounting for in the total fields: less than 5% scored 0; 5% to 25% scored 1; 25% to 50% scored 2; 50% to 75% scored 3; and 75% or more scored 4.

For semiquantitative assessment of C3aR and C5aR, ten arbitrarily chosen 40× objective fields of liver parenchyma in each liver section were examined by light microscopy for the expressions of C3aR and C5aR in liver parenchyma in a blinded fashion. The cumulative scores for each animal were calculated as average the number of positive cells in all visual fields.

Liver tissue samples were stained with hematoxylin and eosin (H&E) to observe liver tissue damage by light microscopy. Semiquantitative assessment of liver injury was performed as reported previously(1).

## Statistical Analysis

## All statistical analyses were performed using the Graphpad Prism Program (version 7.00; GraphPad Software, Inc.). Results at different time groups were analyzed by one-way ANOVA with Dunnett’s post test. The semiquantitative assessment of liver injury were compared using Student’s t test with Welch’s correction.

# Supplementary Figures

## Supplementary Figures Legends

**
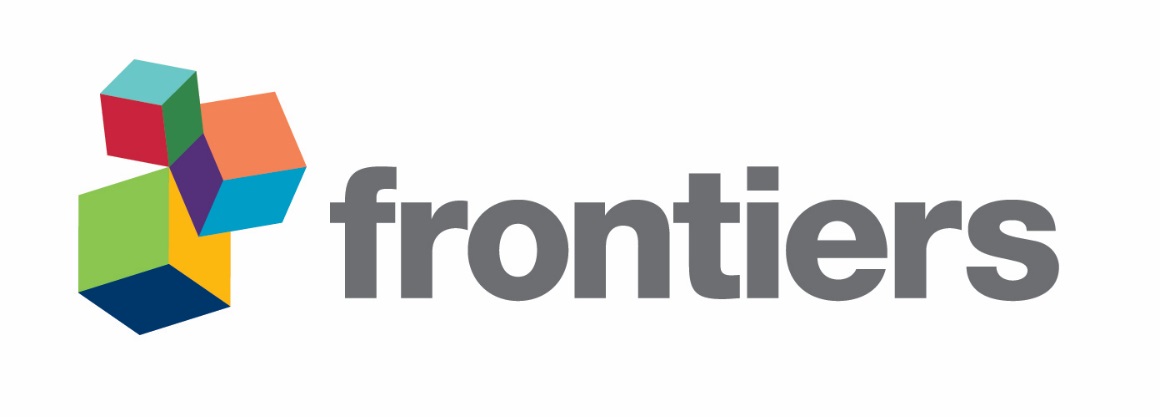
**

**Supplementary Figure 1.** Mice were administered with C3aR antagonist 12 and 36 hrs after MHV-3 infection and euthanized, and liver tissue were collected. (A-B) Macrophage infiltration was detected by immunohistochemical staining 48 hrs after virus infection (red arrow, macrophage). (C) Semiquantitative assessment of macrophage infiltration in livers 48 hrs after virus infection. Representative images are shown (*n* = 5-6 per group). Original magnification, × 200.

**Supplementary Figure 2.** Mice were administered with anti-C5aR antibody 12 hrs after MHV-3 infection and euthanized, and liver tissue were collected. (A-B) Macrophage infiltration was detected by immunohistochemical staining 48 hrs after virus infection (red arrow, macrophage). (C) Semiquantitative assessment of macrophage infiltration in livers 48 hrs after virus infection. Representative images are shown (*n* = 5-6 per group). Original magnification, × 200.

**Supplementary Figure 3.** WT mice were euthanized 0, 12, and 48 hrs after MHV-3 challenge. (A-D) Semiquantitative assessment for the deposition of C3, C5b-9, and for the expression of C3aR and C5aR in liver tissue samples 48 hrs after virus infection. These results are representative of three independent experiments with similar results (*n* = 3-4 per group). ***p*<0.01, *** *p*<0.001, **** *p*<0.0001 compared with WT control.

**Supplementary Figure 4.** WT mice, C3-/- mice, mice administered C3aR antagonist and Mice administered anti-C5aR antibody or the isotype antibody were euthanized 48 hrs after MHV-3 challenge. (A-C) Semiquantitative assessment of livers injury 48 hrs after virus infection by liver damage score. These results are representative of three independent experiments with similar results (*n* = 3-5 per group). * *p*<0.05, ***p*<0.01 compared with WT control.

References:

1. Sun S, Zhao G, Liu C, Wu X, Guo Y, Yu H, et al. Inhibition of Complement Activation Alleviates Acute Lung Injury Induced by Highly Pathogenic Avian Influenza H5N1 Virus Infection. American Journal of Respiratory Cell and Molecular Biology. 2013;49(2):221-30.
