# Supplementary material for: A Dual Role of Complement Activation in the Development of Fulminant Hepatic Failure Induced by Murine-Beta-Coronavirus Infection
Source: Front Cell Infect Microbiol. 2022 Apr 29;12:880915. doi: 10.3389/fcimb.2022.880915 (PMC9099255; doi:10.3389/fcimb.2022.880915)
Supplement: Supplementary file 2 [file Presentation_1.pptx]

## Slide 1
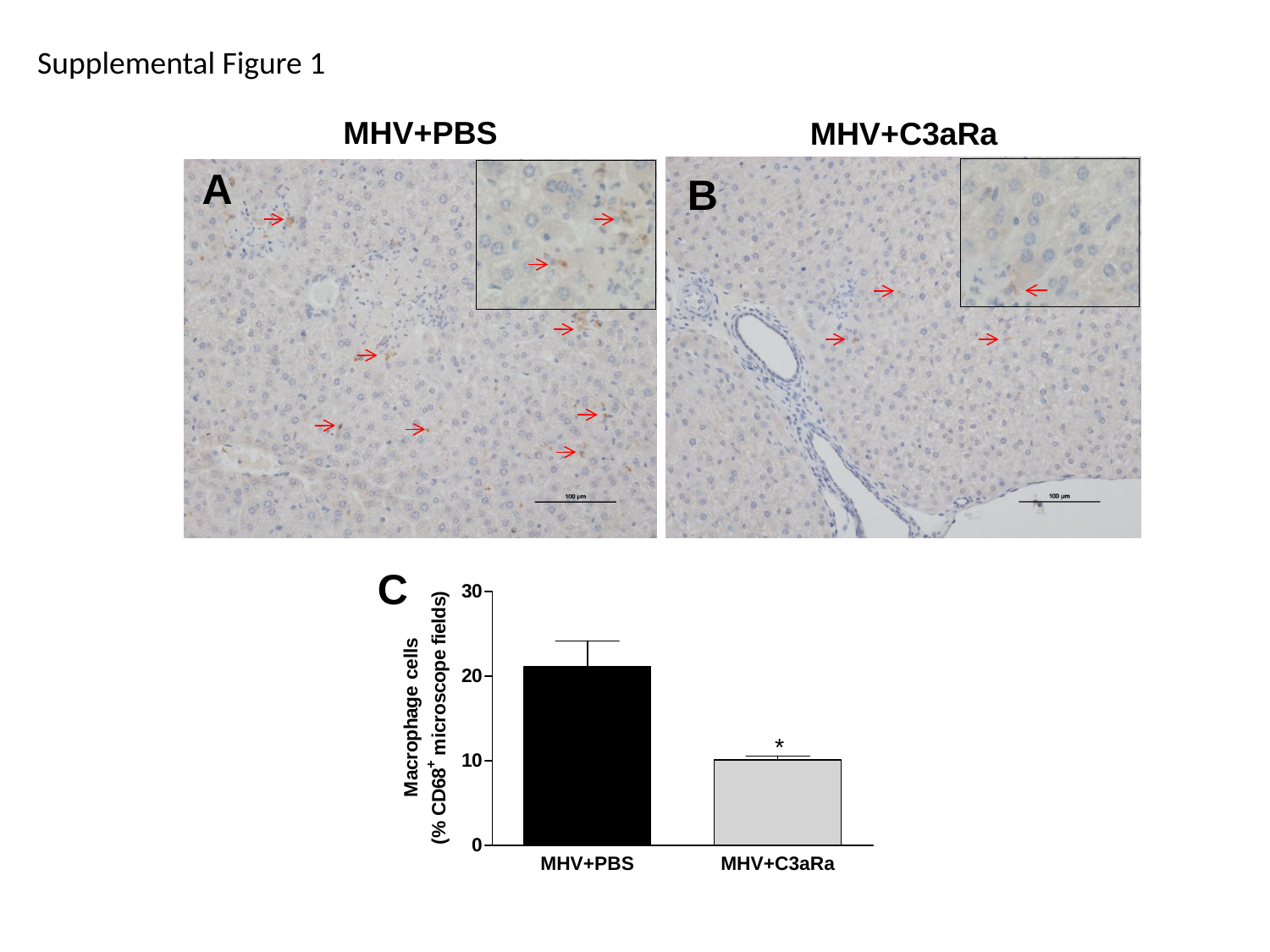

Supplemental Figure 1
MHV+PBS
MHV+C3aRa
A
B
C

## Slide 2
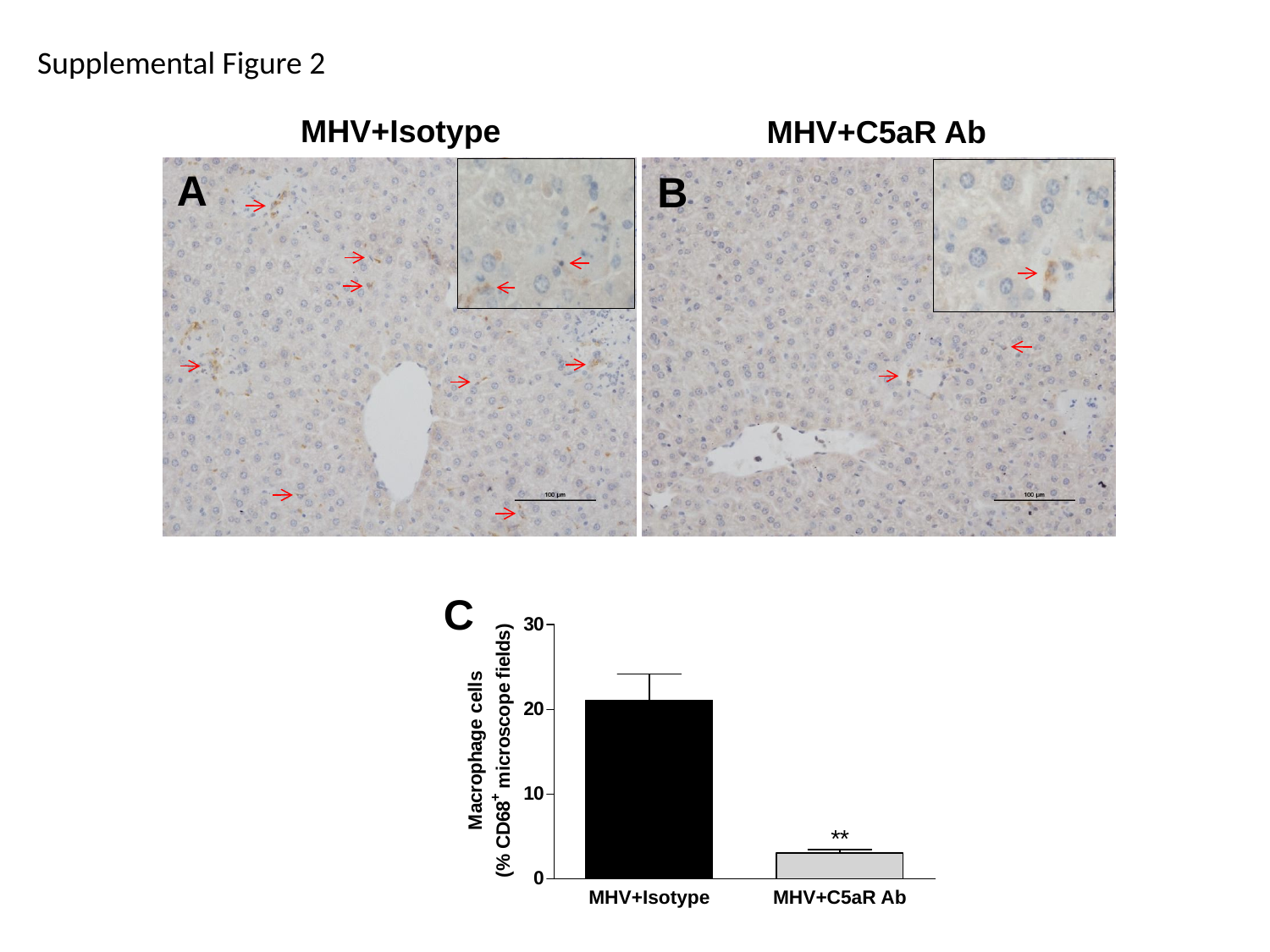

Supplemental Figure 2
MHV+Isotype
MHV+C5aR Ab
A
B
C

## Slide 3
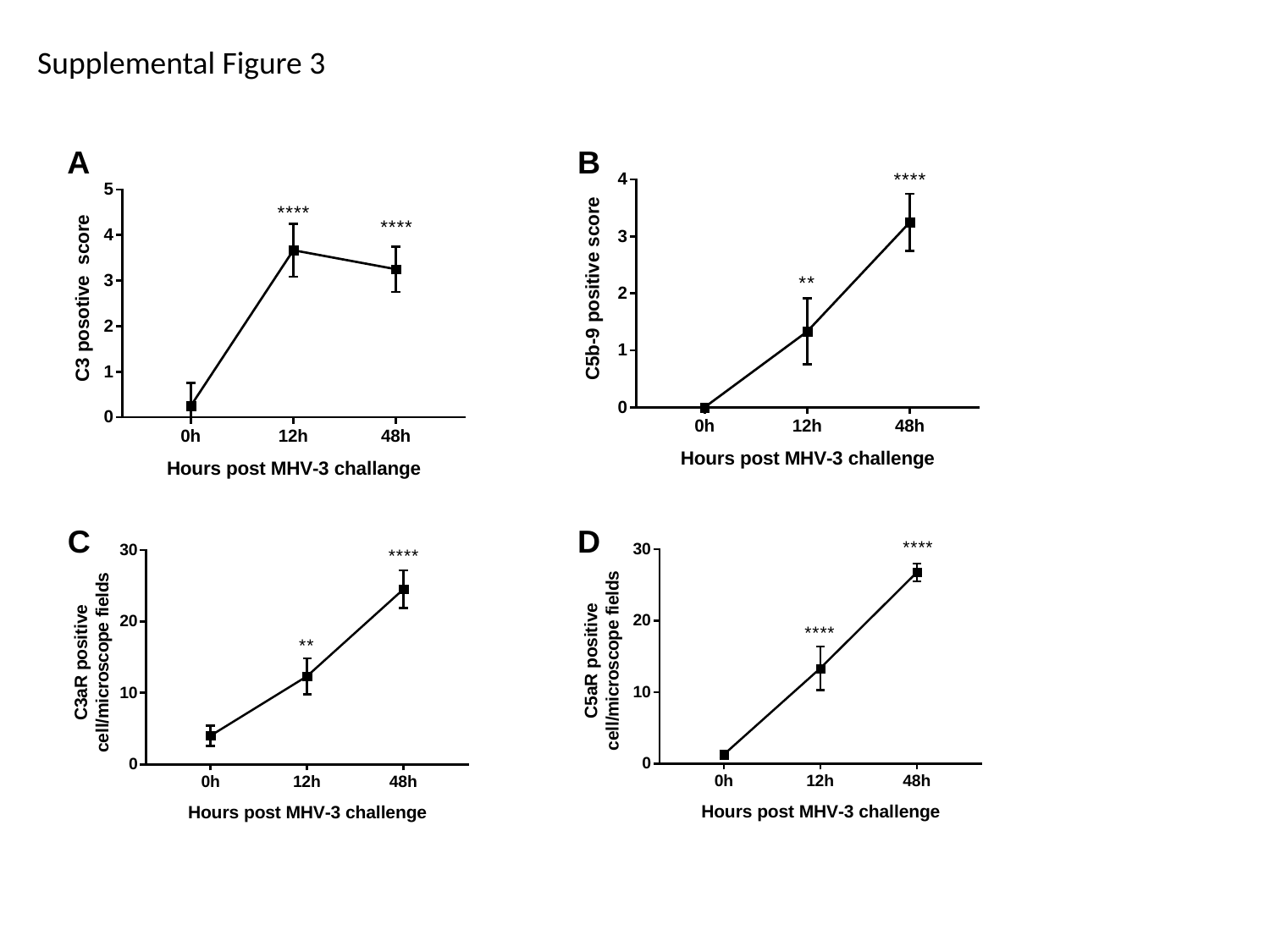

Supplemental Figure 3
A
B
C
D

## Slide 4
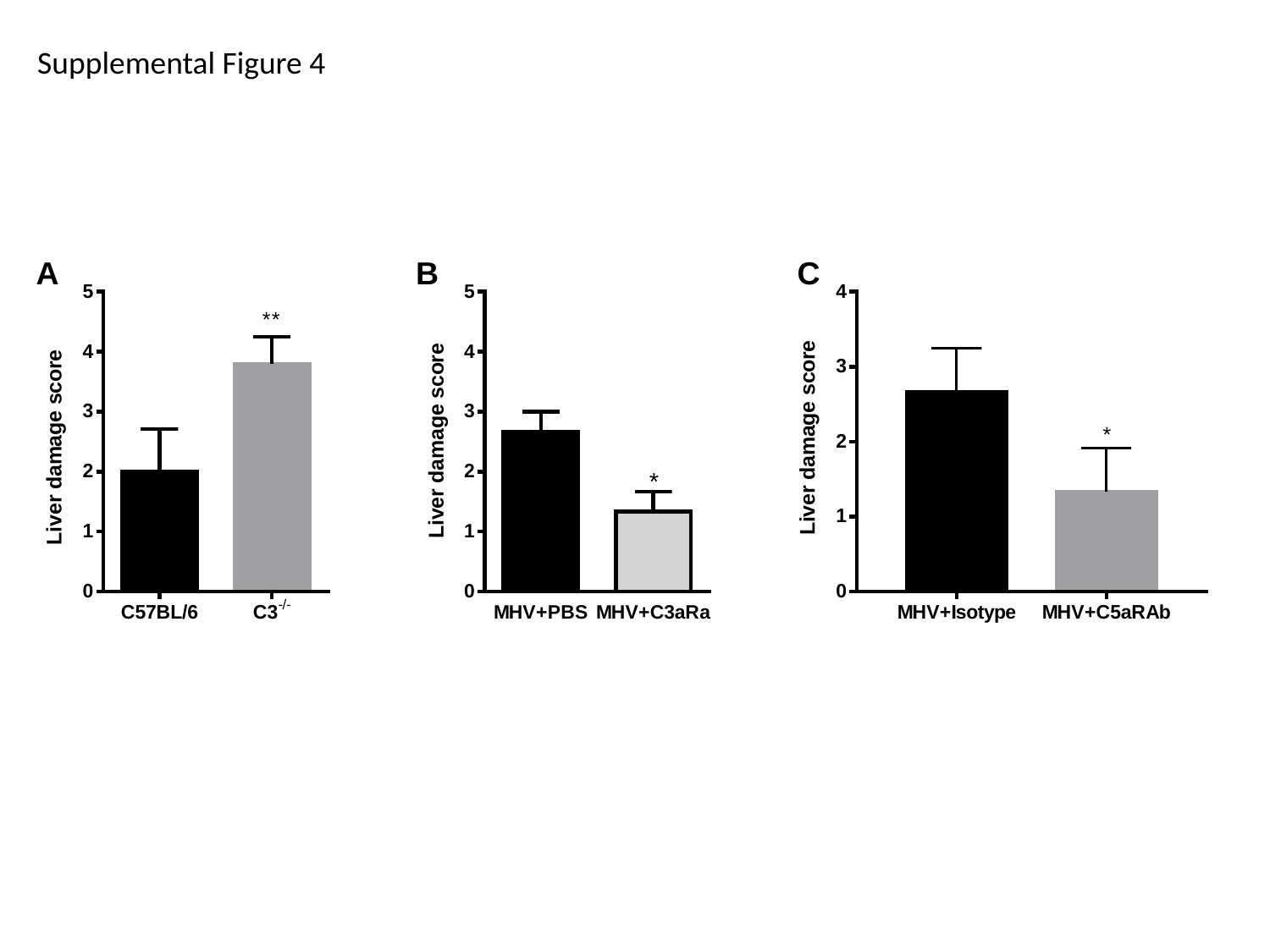

Supplemental Figure 4
A
B
C
